# Europe’s Climate Target for 2050: An Assessment

**DOI:** 10.1007/s10272-021-1012-7

**Published:** 2021-12-11

**Authors:** Richard S. J. Tol

**Affiliations:** Department of Economics, University of Sussex, Sussex House, Falmer, BN1 9SL Brighton, UK

## Abstract

Decarbonisation is harder for transport, heating, industry and agriculture. That is, a doubling of the decarbonisation rate requires much more than a doubling of the policy effort. The low-hanging fruit has been picked.

The European Union has set ambitious targets for greenhouse gas emission reduction. Net emissions should fall to 45% of their 1990 levels by 2030, and to zero by 2050. What are the costs and benefits of this? Do the benefits exceed the costs?

The European Commission has not answered this question. This is unfortunate, as the decision has been made to pursue these goals. The European Commission (2020) has published an Inception Impact Assessment, which is largely qualitative.[1] The in-depth analysis accompanying the Communication for the earlier, less ambitious targets does not report a cost-benefit analysis either (European Commission, 2018), even though the European Commission (2014) has continuously promoted its use. Studies by independent academics find that EU climate policy does not pass the cost-benefit test (Pearce, 2004; Tol, 2007; 2012). However, these studies do not assess the latest plans. This paper fills that gap.

Cost-benefit analysis should not dictate policy. It should inform policy along with other concerns. Yet, economic efficiency is an important criterion. If the costs exceed the benefits, all other policy demands would be harder to meet as there is less money to go around.

This paper reviews the targets set by the European Union, discusses the costs of greenhouse gas emission reduction as well as some political claims about those costs, surveys the benefits of avoided climate change and concludes by comparing costs and benefits, in total and at the margin.

## The scale of Europe's ambition

Figure 1 reveals just how ambitious the EU target really is. Between 1990 and 2019, greenhouse gas emissions fell

1 It does refer to a more detailed report, but that link is dead and the report could not be located by me. Some believe the results of the analysis were "predefined" (Simon, 2020).

**Richard S. J. Tol**, University of Sussex, Falmer, UK; and Vrije Universiteit, Amsterdam, The Netherlands.

Figure 1

**Total greenhouse gas emissions from the 27 member states of the European Union**

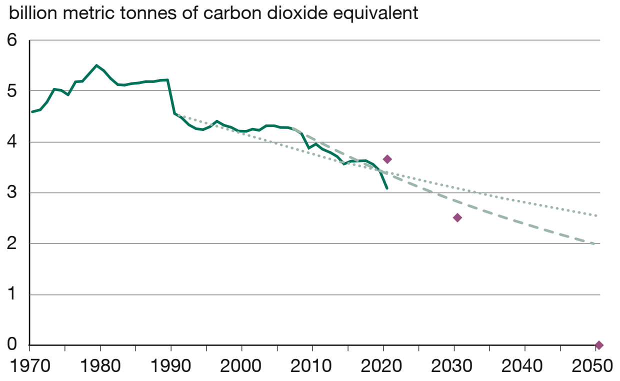

Notes: The solid green line represents the observations, the dotted and dashed light green lines are simple trend projections, the pink diamonds are the targets.

Source: Author's own illustration.

by 1% per year. This accelerated to almost 2% per year between 2007 and 2019.[2] The 2030 target requires that the rate of decarbonisation doubles again, to almost 4% per year. The 2050 target is more ambitious still.

This simple calculation is, in fact, too optimistic. Renewable sources of electricity are one of the key drivers behind the drop in past emissions. In 2019, wind and solar power made up about one-third of all electricity supply (Eurostat, 2021). However, the technical challenges of integrating non-dispatchable sources grow with their share in power generation. Moreover, electricity is probably the easiest sector to decarbonise. Decarbonisation is harder for transport, heating, industry and agriculture. That is, a doubling of the decarbonisation rate requires much more than a doubling of the policy effort. The low-hanging fruit has been picked.

The problems do not stop there. The energy sector, where most of carbon dioxide emissions originate, is characterised by long-lived capital. The year 2050 may seem a long way in the future – six general elections – but a lot of the buildings, power plants, steel mills and chemical plants we use today will still be around in 2050, and even some of the machinery and vehicles (Davis et al., 2010; Tong et al., 2019). That is why the European Union's target is *net* zero. *Gross* zero would require capital destruction at a large scale, with bankruptcies, lay-offs and claims for compensation. Net zero emission requires offsets (emission reduction paid for by the EU but outside the EU), afforestation in Europe (large plantations of rapidly growing trees) or negative carbon energy (electricity generated from biomass with carbon capture or storage). The problem with offsets is that there are few or none if the whole world has a net zero goal. Scale and speed are the problems with afforestation. Agricultural lands are already converting back to nature in Europe. This can be accelerated but not by much. Besides, we prefer diverse forests, including slow-growing species. Scale is also the problem with bioenergy. Cheap biofuel requires large, heavily mechanised mono-plantations. The acreage needed to supply the EU is large, the acreage for the world is unfeasibly large (Wise et al., 2009). The EU strategy for net zero thus seems to bank on the rest of the world not following suit.

## The costs of emission reduction

Emission reduction costs money (Weyant, 1993; Clarke et al., 2014) as climate policy forces people and companies to use different technologies and different fuels than they would have without climate policy. Most studies agree that a complete decarbonisation of the economy could be achieved at a reasonable cost if policies are smart, comprehensive and gradual and if targets are sensible. Models disagree, however, on how much emission reduction would cost. Estimates vary by an order of magnitude or more (Clarke et al., 2014). The main reason is that predicting the future is hard, but modellers could also pay more attention to model calibration and validation (Tol, 2014).

Using the IIASA SSP Database,[3] Tol (2020a) reports that meeting the targets of the Paris Agreement would cost between 0.5% and 10.5% of GDP in 2050, with a model average of some 3%. This would increase to between 1% and 21% in 2100, with a central estimate around 5%. The carbon price would rise to €500/t$CO_2$ in 2050, a price inflation of 24% per year between now and then in the EU,[4] and above €2,000/t$CO_2$ in 2100, a price inflation of 7% per year.

Barker et al. (2007) and Clarke et al. (2009) found that the 2 degrees Celsius target is unfeasible for physical, technical, economic or political reasons. There is a political demand for the analysis of ambitious climate targets, ini-

2 I ignore the sharp drop in emissions in 2020, which is due to the COVID-19 pandemic and unlikely to have a large structural effect on emissions.

3 https://iiasa.ac.at/web/home/research/researchPrograms/Energy/IPCC_AR5_Database.html.

4 The carbon price is higher in the EU than elsewhere, and many countries have no carbon price at all.

tially focused on the 2 degrees Celsius target and more recently on 1.5 degrees Celsius.

Modellers have met that demand by expanding options for negative emissions. This includes negative carbon energy, e.g. biomass with carbon capture and storage (Wise et al., 2009), and direct air capture, e.g. artificial photosynthesis or some other chemical process to remove carbon dioxide from the atmosphere (House et al., 2011).

As the market for carbon dioxide is saturated, negative emissions require a carbon subsidy (and deserve one, as this is a negative externality). Reviewing recent estimates, Tol (2020a) finds that the central estimate of these subsidies amounts to 4% of world income by the end of the century, with one model putting it at almost 17%. Carbon subsidies may thus pose a very substantial burden either on other public expenditure or on taxpayers. Incidence may be politically problematic. Energy crops will be grown in monoculture on large, corporate, heavily mechanised farms in foreign countries. Processing will similarly be done by large firms. An electoral strategy based on large subsidies to agri-energy multinationals is hard to sustain, particularly if negative carbon energy is successful and the threat of climate change recedes.

The cost estimates above assume cost-effective implementation of climate policy. Under ideal conditions, first-best regulation is straightforward: The costs of emission reduction should be equated, at the margin, for all sources of emissions (Baumol and Oates, 1971). Governments routinely violate this principle, with different implicit and even explicit carbon prices for different sectors and for differently sized companies within sectors. Although climate change is a single externality, emitters are often subject to multiple regulations (Boehringer et al., 2008; Boehringer and Rosendahl, 2010). Regulations are often aimed at a poor proxy for emissions (e.g. car ownership) rather than at emissions directly (Proost and Van Dender, 2001). Instrument choice may be suboptimal (Webster et al., 2010), and conditions are not ideal. Optimal policy deviates from the principle of equal marginal costs to accommodate for market power (Buchanan, 1969), for multiple externalities (Ruebbelke, 2003; Parry and Small, 2005) and for prior tax distortions (Babiker et al., 2003). Such deviations are subtle and context specific, and rarely observed in actual policy design. All of this means that *actual* climate policy is far more expensive than what is assumed in models (Boehringer et al., 2009).

Creating jobs is a central part of the political appeal of climate policy in the EU, the UK and the US. Relatively labour-intensive, domestic renewables expand at the expense of more labour-extensive, imported fossil fuels. Job creation in the green economy is partly offset by job destruction in the brown economy.

Furthermore, only a small fraction of the labour force is employed in the energy sector. Changes in the labour intensity of the energy sector therefore cannot have a substantial impact on overall employment. More expensive energy has only a small negative effect on employment in sectors other than energy, but this small proportional effect can, in absolute terms, outweigh the impact in the energy sector as it applies to so many more workers (Patuelli et al., 2005) – unless the revenue of a carbon tax or permit auctions is used to stimulate the economy or reduce the cost of labour (Bovenberg and Goulder, 1996).

Historically, productivity has increased, and wages with it, as capital and energy were used to complement labour. Needing more workers for the same output of energy – the very definition of an increase in the labour intensity of the energy supply – is thus a sign of *regress* rather than *progress*. Baumol's Cost Disease, a rise in wages without a concomitant rise in labour productivity (Baumol and Bowen, 1966), affects renewable energy. Decentralised power generation means decentralised installation, maintenance and retirement of equipment. Technicians thus spend more time travelling and are less productive. Yet, their wages need to compete with those in other sectors of the economy.

## The benefits of emission reduction

### The total impact of climate change

Tol (2018) reviews the 27 published estimates of the total economic impact of climate change, a rather thin basis for any conclusion. The central estimate of the welfare change caused by a century of climate change is comparable to the welfare loss caused by losing a year of economic growth.

Initial warming is positive on net, while further warming would lead to net damages. The initial benefits are due to reduced costs of heating in winter, reduced cold-related mortality and morbidity, and carbon dioxide fertilisation, which makes plants grow faster and more resistant to drought. These initial benefits are sunk, unaffected by current and future emission reduction. For more pronounced warming, the negative impacts dominate, such as summer cooling costs, infectious diseases and rising sea levels.

The uncertainty about the welfare impact of climate change is large and right-skewed. Negative surprises are more likely than positive surprises of a similar magnitude. Feedback that accelerates climate change is more prevalent than feedback that dampens warming, and the impacts of climate change are more than linear in climate change. In that light, the above conclusion needs to be rephrased: A century of climate change is no worse than losing a decade of economic growth.

Estimates are not only uncertain but incomplete too. Some impacts, e.g. on violent conflict, are omitted altogether because they resist quantification. Other impacts are dropped because they do not fit the method such as higher-order impacts in the enumerative method and non-market impacts in computable general equilibrium models. Assumptions about adaptation are stylised, either overly optimistic such as rational agents with perfect expectations in markets without distortions, or overly pessimistic, for example dumb farmers doggedly repeating the actions of their forebears. Valuation of non-market impact is problematic too as benefit transfer, i.e. the extrapolation of observed (or rather inferred) values to unobserved situations, has proven difficult (Brouwer, 2000) but is key to predicting how people will value risks to health and nature in the future.

## The social cost of carbon

The social cost of carbon is the damage done, at the margin, by emitting more carbon dioxide into the atmosphere. If evaluated along the optimal emissions trajectory, the social cost of carbon equals the Pigou tax (Pigou, 1920) that internalises the externality and restores the economy to its Pareto optimum (Pareto, 1906) where no one can be made better off without making someone else worse off. The social cost of carbon also equals the marginal benefit of emission reduction.

The social cost of carbon is a central parameter in the economics of climate change and therefore much estimated and debated. Tol (2021) counts 5,791 estimates in 201 papers, published between May 1982 (Nordhaus, 1982) and April 2021 (Taconet et al., 2021). Table 1 shows the mean and standard deviation of the published estimates and of a fitted kernel density (see Tol, 2021, for details), which better reflects the right-skewed uncertainty about these estimates. The table also splits the sample by the pure rate of time preference, or utility discount rate, used as this is the key driver of the estimates (Anthoff and Tol, 2013).

The sample mean is €42/t$CO_2$, well below €59/t$CO_2$, the emission permit price on 5 November 2021. The social costs of carbon tend to be higher for lower pure rates of time preference. The kernel density assumes that the uncertainty is right-skewed and fat-tailed. The kernel mean is therefore substantially higher than the sample mean. The standard deviation is large relative to the mean, as predicting the future is hard and estimates of the social cost of carbon require many, often controversial assumptions.

Table 1

**Empirical and kernel average of estimates of the social cost of carbon by pure rate of time preference**

in euro per tonne of carbon dioxide

| Pure rate of time preference | Empirical mean (standard deviation) | Kernel mean (standard deviation) |
|---|---|---|
| 3% | 12 (15) | 22 (22) |
| 2% | 74 (160) | 213 (207) |
| 1% | 43 (79) | 115 (139) |
| 0% | 110 (145) | 226 (219) |
| all | 52 (110) | 148 (161) |

Note: The estimates are in 2021 euro per metric tonne of carbon dioxide, for emissions in 2020.

Source: Author's own calculations.

As noted above, the social cost of carbon can be used to put a value on the emissions reduced. The European Union, however, expresses its emission reduction targets in percentages of 1990 emissions, rather than in tonnes of carbon dioxide not emitted. Figure 1 gives a rough idea of emissions avoided. In 2030, depending on the scenario, between 317 and 577 million tonnes of carbon dioxide equivalents would not be emitted. This increases to between 1,978 and 2,535 $MMTCO_2eq$ in 2050. If the social cost of carbon increases by 2.2% per year, the EU's planned emission reduction is worth between €29 and €53 billion in 2030 and between €283 and €363 in 2050.

## The benefits of climate policy

Instead of using the marginal benefits to approximate the impact of EU climate policy, implicitly assuming linearity, we can also estimate this directly. Tol (2018) fits seven alternative damage functions to the 27 published estimates of the total economic impact of climate change. Figure 2 shows the weighted average of these damage functions, using the relative likelihoods as weights. Two scenarios are shown: no (additional) policy and current pledges (see Ou et al., 2021). The horizontal axis is time, the vertical axis the Hicksian equivalent variation, i.e. the change in income that would make the average person feel as unhappy as they would about climate change.

There is no discernible difference between the two scenarios in 2030. The momentum in emissions, concentrations, atmosphere and ocean is simply too large to expect much if anything within a decade. By 2050, the "no policy" scenario shows a negative impact on climate change (at

Figure 2

**The global total annual impact of climate change for two alternative policy scenarios**

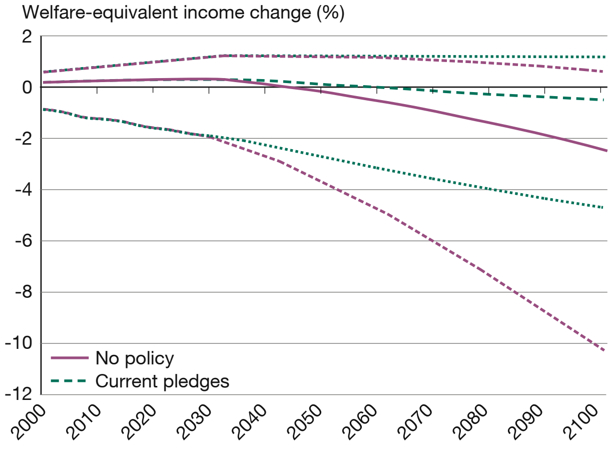

Source: Author's own calculations.

least in the central estimate) while the "current pledges" scenario still has positive impacts on climate change. The difference is 0.3% of world income or, for today's output, some €220 billion.

## Discussion and conclusion

The numbers reviewed above are sobering. The total cost of greenhouse gas emission reduction could be 3% or more of GDP. The benefits would be only 0.3% of GDP, a benefit-cost ratio of one in ten. The marginal costs and benefits give the same message. The marginal costs of greenhouse gas emission reduction would reach €500/t$CO_2$ by 2050 while the marginal benefits would be less than €150/t$CO_2$, a benefit-cost ratio of three in ten.

It is often argued that the impacts of climate change are underestimated. Impact estimates are certainly incomplete (Arent et al., 2014). However, arguing that the impacts are off by a factor of ten or even a factor of three is quite a stretch. In fact, the percentage above is the *global* average; a rich region such as Europe would be less vulnerable (Tol, 2018). The social cost of carbon is the *global* social cost of carbon; the EU social cost of carbon would be a fraction of this (Tol, 2019).

Besides, the costs of climate policy are underestimated too, based on the rather unrealistic assumptions of a first-best implementation in an economy without other distortions. In reality, we observe a jumble of policies, uncoordinated not just between countries but within countries as well, and sharp shifts over time as political whims and electoral fortunes come and go.

That said, the above estimates assume stringent climate policy outside the EU too. If climate policy elsewhere were more lenient, then the costs of greenhouse gas emission reduction in Europe would be lower as there would be less competition on the markets for renewables and offsets. At the same time, the benefits of climate policy would be larger. While this would improve the benefit-cost ratio, it is unlikely to make a factor of three, let alone ten difference.

It is therefore safe to conclude that the benefits of the European Union's climate policy do not outweigh its costs. There are no immediate political implications of this finding. The European Union has put stringent emission targets front and centre of its entire policy agenda. There is little political opposition. However, in the longer term, the stringent targets are vulnerable as the costs and other implications of meeting them become apparent to a growing number of people. As climate continues to change, it will also become clear that the weather disasters foretold will not have materialised. At that point, public and political support for the EU's climate policy will likely crumble, and result in a tax revolution as predicted by Dowlatabadi (2000) and observed with the *gilets jaunes* in France in 2018.

Further research is needed on all aspects of climate policy. I do not expect much progress on the economic impacts of climate change, not until the literature gets itself out of the rabbit hole of confusing weather shocks and climate change, despite previous warnings not to (e.g. Dell et al., 2014). More progress can be expected from the new empirical literature on the costs of greenhouse gas emission reduction, the somewhat belated realisation by economists that climate policy started in 1991 and can be studied *ex post* as well as the more common *ex ante*. The resulting papers suggest that climate policy is more difficult and expensive than is commonly assumed (Leahy and Tol, 2012; Fowlie et al., 2018; Lin and Wesseh, 2020; Runst and Thonipara, 2020). Yet more progress lies in the study of second-best climate policy, with studies revealing again higher policy costs (Barrage, 2020; Tol, 2020b).

Until research has progressed, the conclusion remains that the costs of EU climate policy far exceed the benefits.
